# Neuregulin 1 Improves Glucose Tolerance in db/db Mice

**DOI:** 10.1371/journal.pone.0130568

**Published:** 2015-07-31

**Authors:** Gaël Ennequin, Nathalie Boisseau, Kevin Caillaud, Vivien Chavanelle, Monique Etienne, Xinyan Li, Pascal Sirvent

**Affiliations:** 1 Université Clermont Auvergne, Université Blaise Pascal, EA 3533, Laboratoire des Adaptations Métaboliques à l’Exercice en Conditions Physiologiques et Pathologiques (AME2P), BP 80026, F-63171, Aubière Cedex, France; 2 CRNH-Auvergne, Clermont-Ferrand, F-63001, France; 3 Zensun Sci & Tech Ltd., Shanghai, China; Georgia Regents University, UNITED STATES

## Abstract

*In vitro* experiments using rodent skeletal muscle cells suggest that neuregulin 1 (NRG1) is involved in glucose metabolism regulation, although no study has evaluated the role of NRG1 in systemic glucose homeostasis. The purpose of this study was to investigate the effect of chronic and acute NRG1 treatment on glucose homeostasis in db/db mice. To this aim, glucose tolerance tests were performed in 8-week-old male db/db mice after treatment with NRG1 (50μg.kg^-1^) or saline 3 times per week for 8 weeks. In other experiments, glucose tolerance and pyruvate tolerance tests were performed in db/db mice 15 minutes after a single NRG1 (50μg.kg^-1^) or saline injection. Liver, adipose tissue, hypothalamus and skeletal muscle were also collected 30 minutes after acute NRG1 (50μg.kg^-1^) or saline treatment, and the phosphorylation status of the ERBB receptors, AKT (on Ser473) and FOXO1 (on Ser256) was assessed by western blotting. Chronic treatment (8 weeks) with NRG1 improved glucose tolerance in db/db mice. Acute treatment also lowered glycemia and insulinemia during glucose or pyruvate tolerance tests. NRG1 acute injection induced activation of ERBB3 receptors and phosphorylation of AKT and FOXO1 only in liver. Altogether, this study shows that acute and chronic NRG1 treatments improve glucose tolerance in db/db mice. This effect could be mediated through inhibition of hepatic gluconeogenesis.

## Introduction

Neuregulins belong to the large epidermal growth factors (EGF) family of proteins. They are four structurally related genes (NRG1-4) that encode many different isoforms characterized by the presence of an EGF-like domain that mediates their biological activity and binding to the v-erb-b2 avian erythroblastic leukemia viral oncogene homologs 3 and 4 (ERBB3 and 4) [1, 2]. NRG1 binding induces homo- or hetero-dimerization of ERBB receptors, leading to the activation of downstream phosphorylation cascades [2–6]. NRG1 isoforms play essential roles in growth, differentiation and survival of nerve, cardiac and skeletal muscle tissues [2, 7–9]. For instance, mice lacking the NRG1 isoforms containing the cysteine-rich domain (CRD) die soon after birth due to inability to breathe and display altered neuromuscular junction formation [10]. Targeted disruption of the NRG1 isoforms containing the immunoglobulin (Ig)-like [11] or the EGF-like domain [12], or deletion of ERBB3 [13] or ERBB4 [14] receptors causes embryonic lethality with cardiac malformation. Therefore, the NRG1/ERBB pathway is considered a potential therapeutic target for the treatment of heart and neuromuscular diseases.

Some studies suggest that the NRG1 pathway is also involved in the regulation of glucose metabolism. Indeed, acute treatment with NRG1 induces translocation of the GLUT4 glucose carrier to the membrane and stimulates glucose uptake in both L6E9 muscle cells and rat soleus [15]. NRG1 action on glucose uptake is mediated by a signaling cascade involving the PI3K-PDK1-PKCζ pathway and this effect is additive to that of insulin [16]. NRG1-induced glucose uptake has also been observed in adult rat ventricular myocytes and was associated with ERK1/2 phosphorylation at Thr202/Tyr204 and AKT phosphorylation on Ser473 [17]. The finding that NRG1-induced AKT phosphorylation at Ser473 can be blunted by pre-treating cardiac myocytes with palmitate [18] suggests that over-nutrition or hyperlipidemia could lead to NRG1 resistance. Besides these acute short-term effects, incubation with NRG1 for 48h increases GLUT4 content in L6E9 muscle cells, suggesting that chronic NRG1 treatment could regulate muscle glucose metabolism and consequently systemic glucose homeostasis [19]. Conversely, NRG1 effects in liver, which also has a key role in glucose metabolism regulation [20], have been less investigated. It has been reported that ERBB3 and, to a less extent, ERBB4 are expressed in liver of adult rats [21–23]. Acute exposure of rat hepatocytes to NRG1 induces ERBB3 phosphorylation and activation of DNA synthesis [24]. Insulin seems to inhibit NRG1 action in rat hepatocytes [24]. In two animal models of insulin deficiency (type I diabetes and fasting), liver ERBB3 expression is repressed following insulin treatment, suggesting an interaction between insulin and the NRG1/ERBB pathway [23].

To date, despite the promising results obtained in skeletal muscle cells *in vitro* [15, 16, 19, 25], no study has evaluated the impact of NRG1 on glucose metabolism *in vivo*. Based on these *in vitro* findings, we hypothesized that NRG1 treatment could improve glucose homeostasis *in vivo* mainly through an effect on skeletal muscle, but possibly also in other tissues that are involved in glucose homeostasis regulation and express NRG1 receptors (liver, adipose tissue and hypothalamus). Such effect could be particularly relevant in the presence of insulin resistance and impaired glucose tolerance, such as type 2 diabetes. We thus tested this hypothesis by evaluating the effects of chronic and acute NRG1 treatment on the systemic glucose metabolism regulation in db/db mice, an animal model of type 2 diabetes.

## Materials and Methods

### Animals

Animal husbandry and experimental procedures were carried out in accordance with the EU Directive 2010/63/EU for animal experimentation and were approved by the local ethics committee for animal testing (CEMEA Auvergne, CE1-09). Two month/old, BKS(D)-*Lepr* ^*db*^ */*JOrlRj (db/db) male mice, provided by CERJ Janvier (Le Genest Saint Isle, France), were kept in temperature-controlled cages (20–22°C) with a reversed light-dark cycle (8pm-8am) and with free access to water and food. At this age, db/db mice are already obese with elevated plasma insulin and glucose. At the beginning of the study, their serum insulin level was 2.14 ± 0.43 ng.ml^-1^ and blood glucose level was 294.4± 19.6 mg.dl^-1^. Both acute and chronic treatment started at this age (2 month/old).

### Chronic treatment with NRG1

For chronic treatments, 50 μg/kg body weight of recombinant NRG1 (Zensun, Shanghai, China, n = 8), or an equal volume of 0.9% NaCl (saline, n = 8), was administered to db/db mice by intra-peritoneal (i.p.) injection three times per week for eight weeks. Treatments were stopped three days before euthanasia. Body weight and food intake were recorded every week during the treatment period.

### Acute effect of NRG1 on basal glycemia

Recombinant NRG1 (50μg/kg body weight, n = 8) or an equal volume of 0.9% NaCl (n = 8) was i.p. injected in fasted (for 6h) db/db mice and blood samples were collected from the tail at 0 (baseline), 15, 30, 60, 90 and 120 minutes after injection. Blood samples were centrifuged at 10 000g for ten minutes and serum samples were stored at -80°C.

### Glucose and Pyruvate Tolerance Tests (GTT and PTT)

After 6h of fasting, a glucose load (2g/kg of bodyweight) or a pyruvate load (1g/kg of bodyweight) was administered by gavage or by i.p. injection, respectively. In some experiments, fasted mice received an injection of 50 μg/kg body weight of recombinant NRG1 (Zensun, Shanghai, China) (n = 8) or an equal volume of 0.9% NaCl (n = 8) fifteen minutes before the glucose/pyruvate load. Blood samples were collected from the tail at 0 (baseline), 30, 60, 90 and 120 minutes after the glucose/pyruvate loads.

### Metabolic measurements

ELISA Assay Kits were used to assess serum insulin (ALPCO Diagnostics, Salem, USA) and glucose levels (BioMérieux SA, Marcy l’Etoile, France). Blood lactate levels were immediately analyzed with an electrochemical single-use strip method (Lactate Pro KDK Corp, Kyoto, Japan).

### Animal dissection

Mice were anesthetized with isofluorane and euthanized by decapitation after 6h of fasting. Gastrocnemius, liver, epididymal white adipose tissue (WAT) and hypothalamus were dissected, weighed, frozen in liquid nitrogen and stored at -80°C for later biochemical analysis. The gastrocnemius muscle was chosen because it presents a “mixed” metabolic and contractile phenotype (both type I and type II fibers) and thus it is more representative of the skeletal muscle tissue than other muscles with more specific profiles. In some experiments, mice received an i.p. injection of recombinant NRG1 (50μg/kg body weight, n = 8) or an equal volume of saline solution (0.9% NaCl, n = 8) thirty minutes before sacrifice.

### Protein extraction

Gastrocnemius and liver (50 mg) were homogenized in 400μl lysis buffer (20 mM Hepes, 350 mM NaCl, 20% (v/v) glycerol, 1% (v/v) Nonidet P-40, 1 mM MgCl_2_, 0.5 mM EDTA, 0.1 mM EGTA, pH 7.9) supplemented with a protease inhibitor cocktail (Sigma-Aldrich Co.) using a Potter-Elvehjem tissue grinder placed on ice. Homogenates were then centrifuged at 10 000g for 5 min and supernatants stored at -80°C for further analysis. Protein content was measured by using the Bradford method (BioRad Laboratories) and BSA as standard (Sigma-Aldrich Co.). Homogenates were diluted with lysis buffer to a final concentration of 30μg/ml.

### Western blotting

Protein samples were diluted with Laemmli buffer, separated on Criterion Stain-Free precast gels in a BioRad Mini PROTEAN Tetra-Cell unit and transferred to nitrocellulose membranes using a BioRad Trans Blot Turbo transfer. Membranes were blocked with 5% nonfat dry milk in Tris buffered saline (pH 7.5) containing 0.1% Tween 20 (TBST) at room temperature for 1h. Then, membranes were incubated in 2% BSA with the relevant primary antibodies at 4°C overnight. Anti-AKT (1/1000), -p-AKT Ser473 (1/1000), -FOXO (1/1000), -p-FOXO, (1/1000), -ERBB3 (1/200) and–p-ERBB3 (1/200) antibodies were purchased from Cell Signaling (Beverly, MA). Anti-ERBB-2 (1/200), -ERBB-4 (1/200), -p-ERBB-2 (1/200), -p-ERBB4 (1/200) and -NRG1 N120A/9 antibodies were purchased from Santa Cruz (Santa Cruz Biotechnology, CA). After incubation with the appropriate horseradish peroxidase-conjugated secondary antibody in TBST at room temperature for 1 hour, enhanced chemiluminescence (ECL) reagents (Pierce) were used to detect interactions and digital images were acquired using the Molecular Imager ChemiDoc XRS System (Biorad). Signals were quantified using the Image Lab 4.1 software (BioRad) and normalized using the Total Protein Normalization (TPN) method provided by Stain Free technology.

### Statistical analysis

The SPSS Advanced Statistics software was used for statistical analysis (IBM). Data are presented as the mean ± SEM. Two-way repeated measures ANOVA was performed, when appropriated, with Tukey's post-hoc analysis. The trapezoidal rule was used to determine the net incremental area under the curve (net AUC, representing the variation from baseline over the duration of the test). The unpaired Student’s *t* test was used to compare two groups.

## Results

### Chronic NRG1 treatment improves glucose clearance in db/db mice

**Fig 1:**
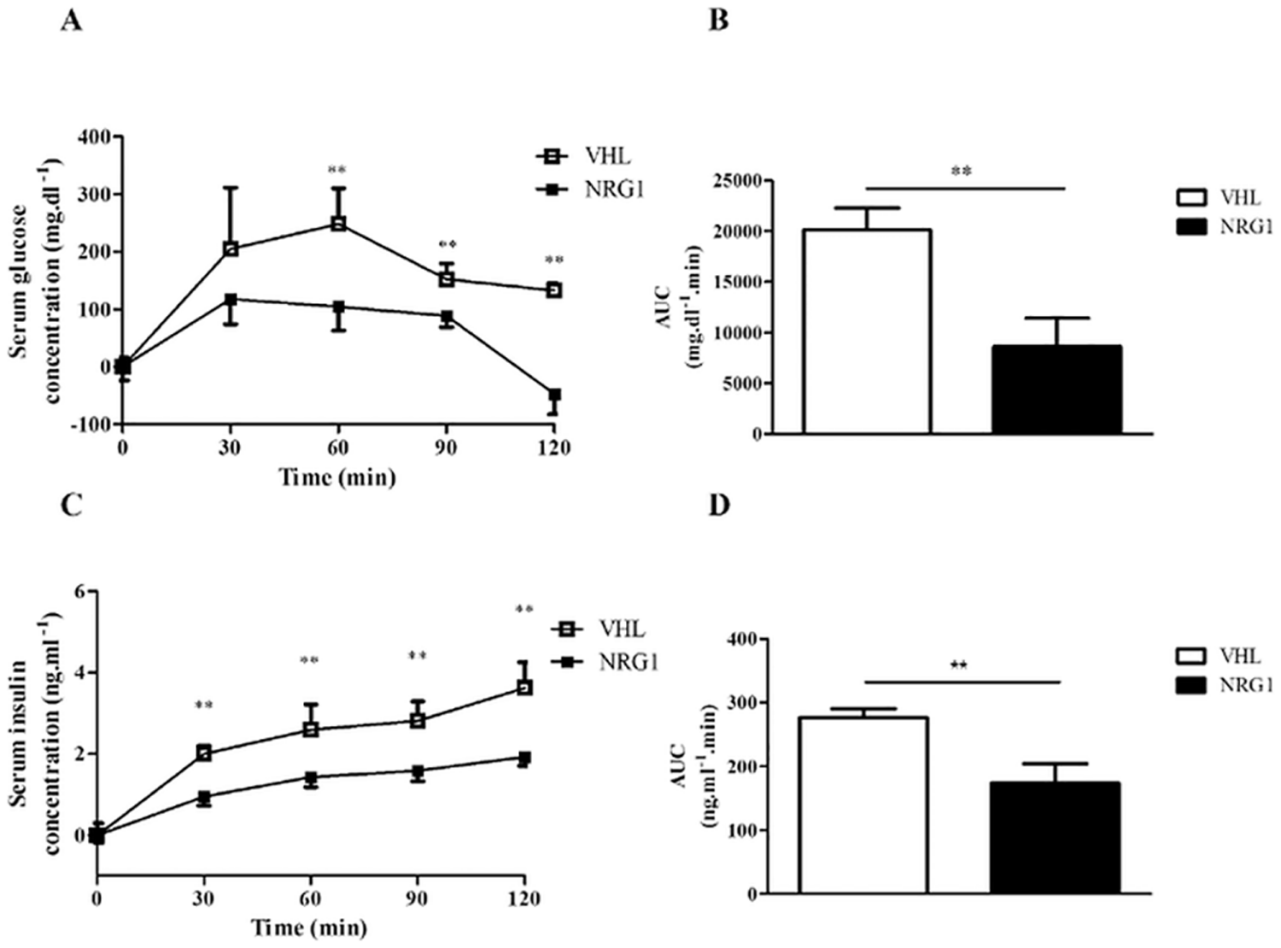
Effects of eight-week treatment with NRG1 on serum glucose and insulin levels during OGTT in db/db mice. Male db/db mice were treated with vehicle (0.9% NaCl; n = 8) or with NRG1 (50 μg.kg^-1^; n = 8) 3 days/week, for eight weeks. Then, mice fasted for six hours were subjected to glucose tolerance test (GTT) by oral gavage of glucose (2.0 g.kg^-1^ of body weight). Blood was sampled at 0 (baseline), 30, 60, 90 and 120 minutes for glucose and insulin measurements. A) Changes in serum glucose level during the GTT relative to basal glycemia. B) The net area under the curve (AUC) was calculated for the data presented in A by using the trapezoidal rule. C) Changes in serum insulin concentration during the GTT relative to basal insulinemia. D) The net AUC was calculated for the data presented in C using the trapezoidal rule. Values are the mean ± SEM. VHL: vehicle; NRG1: neuregulin 1; **: p<0.01 compared to VHL.

We first evaluated the effect of chronic treatment (8 weeks) with NRG1 (n = 8) or saline (vehicle, VHL, n = 8) on glucose metabolism in db/db mice. The mode and frequency of NRG1 administration (i.p. injections, 3 times per week) was chosen based on the protocol used to test NRG1 effect on the muscle phenotype of mdx mice [26]. At the end of the treatment period, our results showed that chronic treatment did not affect body weight (VHL: 46.6 ± 1.3g and NRG1: 45.8 ± 1.9g) and food intake (VHL: 42.8 ± 3.3g.week^-1^ and NRG1: 42.3 ± 1.9g.week^-1^). The basal serum concentrations of glucose and insulin were not significantly different between NRG1- and VHL-treated mice (glucose: 491.7 ± 30.4 vs 545.7 ± 11.4 mg.dl^-1^ and 1.39 ± 0.18 vs 1.25 ± 0.29 ng.ml^-1^, respectively; data not shown). Conversely, serum glucose concentration at the different time points of the GTT was lower in db/db mice treated with NRG1 compared with controls (VHL) (Fig 1A). Accordingly, the net AUC, which represents the variation in glucose concentration from baseline over the test duration, was significantly smaller in NRG1-treated than in VHL-treated mice (8620 ± 2783 vs 20153 ± 2134 mg.min.dl^-1^, respectively, p <0.01) (Fig 1B). The concomitant analysis of serum insulin concentration during GTT also showed a lower increase in the NRG1 than in the VHL group (Fig 1C) with a significantly smaller net AUC in the NRG1 group than in control animals (174.1 ± 30.1 vs 276.5 ± 13.7 mg.min.dl^-1^, p <0.01; Fig 1D).

### Acute NRG1 treatment reduces serum glucose concentration in db/db mice

**Fig 2:**
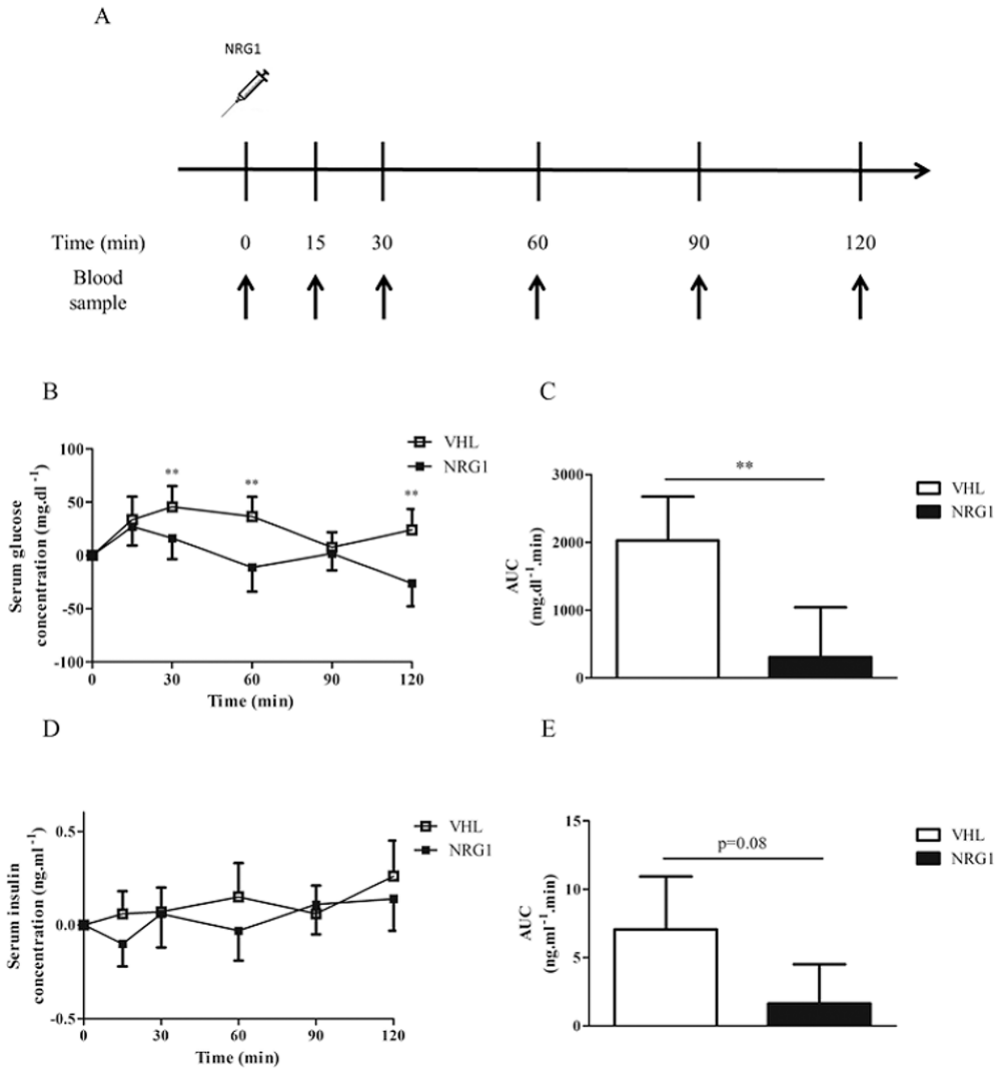
A single NRG1 injection lowers serum glucose in db/db mice. A) Male db/db mice fasted for six hours received an i.p. injection of vehicle (0.9% NaCl; n = 8) or NRG1 (50 μg.kg^-1^; n = 8) and blood was sampled at different time-points. B) Changes in serum glucose concentration following NRG1 or vehicle injection relative to basal glycemia. C) Net AUC calculated for the data presented in B by using the trapezoidal rule. D) Changes in serum insulin concentration following NRG1 or vehicle injection relative to basal insulinemia. E) Net AUC calculated for the data presented in C using the trapezoidal rule. Values are the mean ± SEM. VHL: vehicle; NRG1: neuregulin 1; **: p<0.01 compared to VHL.

To further investigate NRG1 role in glucose metabolism, we evaluated, in fasted db/db mice, the effect of a single injection of NRG1 (50μg.kg^-1^, n = 8) or saline (VHL, controls, n = 8) on serum glucose concentration over two hours (Fig 2A). In VHL-treated mice, glycemia slightly increased, particularly during the first 30 minutes, possibly due to the stress caused by the experiment. Conversely, serum glucose concentration significantly decreased in NRG1-treated mice, compared with controls (Fig 2B), resulting in a reduction of the net AUC area by 85% (Fig 2C). Acute NRG1 treatment did not have any significant effect on serum insulin levels (Fig 2D and 2E).

### Acute NRG1 treatment reduces serum glucose and insulin concentrations in db/db mice during an oral glucose tolerance test

**Fig 3:**
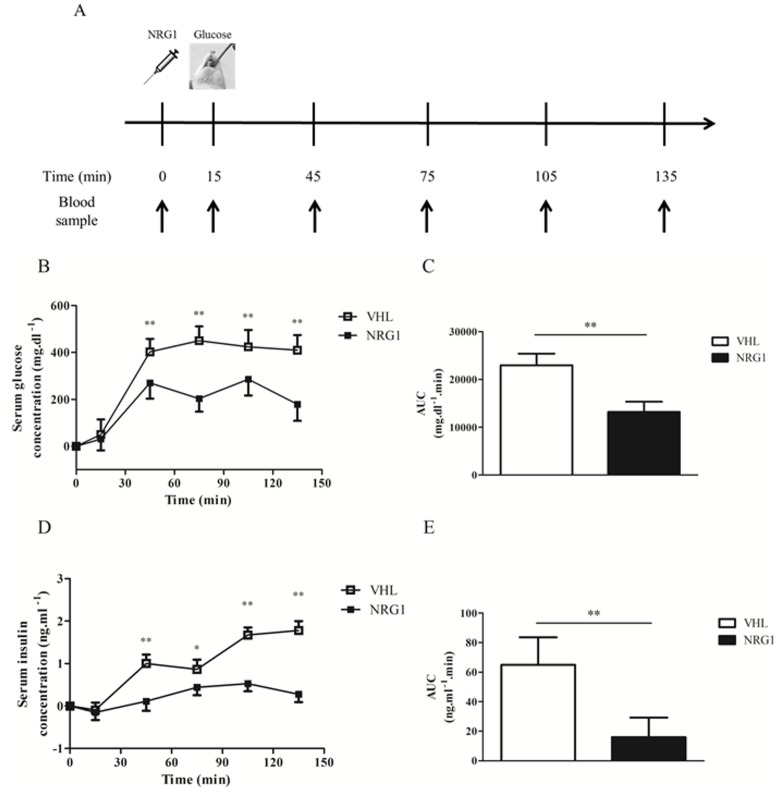
A single NRG1 injection lowers the serum glucose and insulin responses to OGTT. A) Six hour-fasted male db/db mice received an i.p. injection of vehicle (0.9% NaCl; n = 8) or NRG1 (50 μg.kg^-1^; n = 8) fifteen minutes before a glucose load (2.0 g.kg^-1^ of body weight). Blood was sampled at the time of the NRG1 injection (0; baseline) and then at 15, 45, 75, 105 and 135 minutes post-injection. B) Changes in serum glucose concentration during the oral glucose tolerance test (GTT) relative to basal glycemia. C) Net AUC calculated for the data presented in B using the trapezoidal rule. D) Changes in serum insulin concentration changes at each point of the GTT relative to basal insulinemia. E) Net AUC for the data presented in C calculated using the trapezoidal rule. Values are the mean ± SEM. VHL: vehicle; NRG1: neuregulin 1;*: p<0.05; **: p<0.01 compared to VHL.

To assess the effect of acute NRG1 treatment on glucose tolerance, which is impaired in db/db mice, NRG1 (50μg.kg^-1^, n = 8) or saline solution (VHL, n = 8) was injected in fasted db/db mice fifteen minutes before a glucose load (2g.kg^-1^) and serum glucose and insulin were measured at different time points over 135min (Fig 3A). In NRG1-treated mice, the increase in serum glucose concentration was significantly lower than in the VHL group (Fig 3B). Accordingly, the net AUC was reduced by 43% in NRG1-injected mice compared with controls (p<0.01; Fig 3C). Serum insulin concentration during the GTT was also significantly reduced in the NRG1 group compared with the VHL group (Fig 3D and 3E).

### Acute NRG1 treatment affects gluconeogenesis in db/db mice

**Fig 4:**
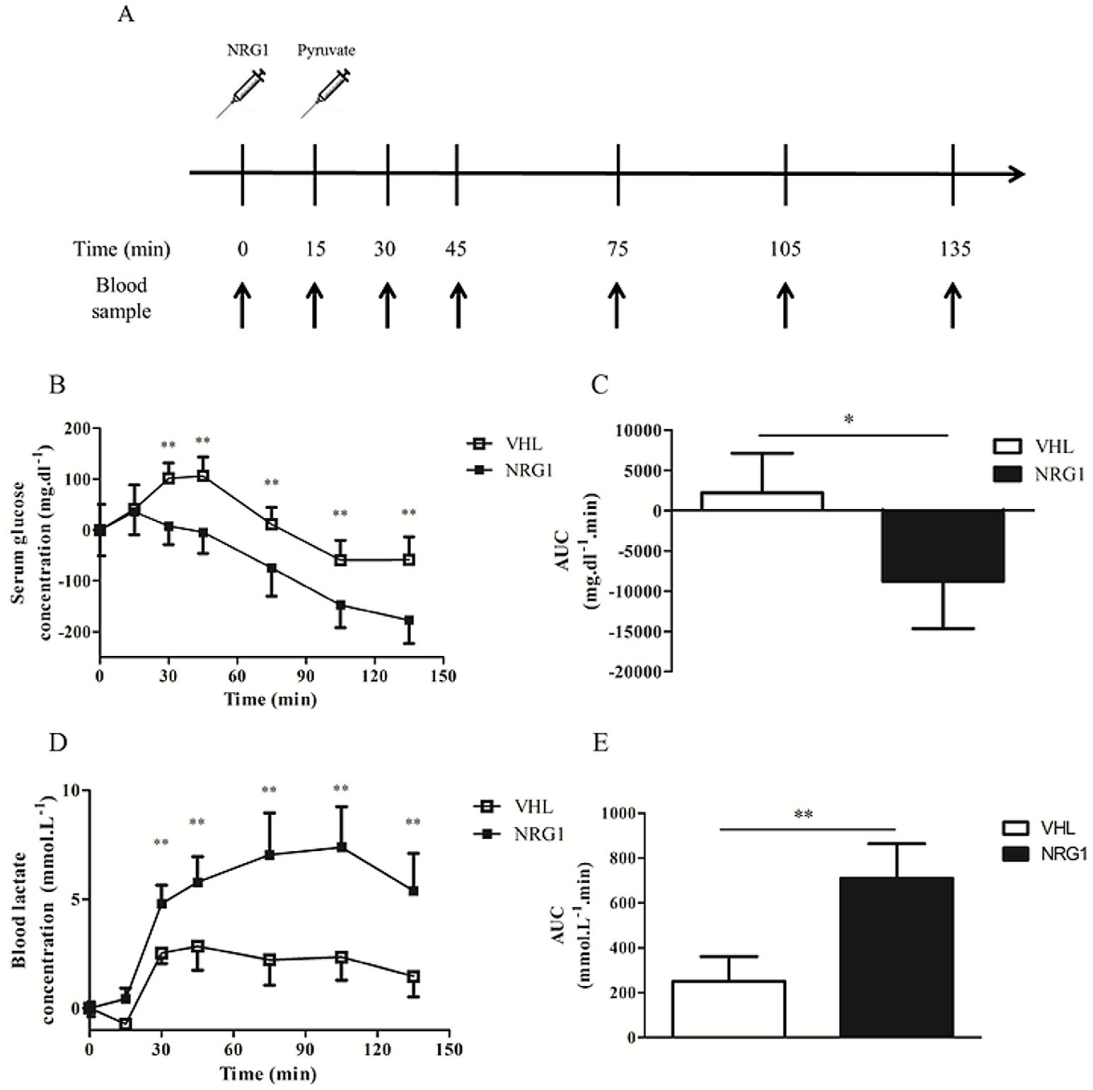
A single NRG1 injection lowers glucose and increases lactatemia during a pyruvate tolerance test. A) Six hour-fasted male db/db mice received vehicle (0.9% NaCl; n = 8) or NRG1 (50 μg.kg^-1^; n = 8/group; NRG1) by i.p. injection fifteen minutes before i.p. injection of 1.0g.kg^-1^ body weight pyruvate. Blood was sampled at the time of the NRG1 injection (0) and then at 15, 45, 75, 105 and 135 minutes post-injection. B) Changes in serum glucose concentration during the pyruvate tolerance test (PTT) relative to basal glycemia. C) Net AUC calculated for the data presented in B using the trapezoidal rule. D) Changes in blood lactate concentration during the PTT relative to basal lactatemia. E) Net AUC calculated for the data presented in D using the trapezoidal rule. Values are the mean ± SEM. VHL: vehicle; NRG1: neuregulin 1;*: p<0.05; **: p<0.01 compared to VHL.

Then, the Pyruvate Tolerance Test (PTT) was used to assess the effect of acute NRG1 administration on glucose production via gluconeogenesis. NRG1 (50μg.kg^-1^, n = 8) or saline (VHL, n = 8) was injected in fasted db/db mice fifteen minutes before the pyruvate load (1g.kg^-1^) and then serum glucose and blood lactate levels were assessed over 135min (Fig 4A). In the VHL group, glycemia transiently increased following pyruvate injection, reflecting the activation of gluconeogenesis in fasted animals. Conversely, in the NRG1 group, glucose levels progressively and significantly decreased, suggesting that gluconeogenesis from pyruvate was inhibited by NRG1 (Fig 4B and 4C). Pyruvate accumulated in cells is typically converted into lactate by lactate dehydrogenase (LDH), resulting in increased lactate concentration. Accordingly, blood lactate levels increased following pyruvate injection (Fig 4D and 4E), particularly in the NRG1 group, further supporting the hypothesis that NRG1 may inhibits pyruvate conversion to glucose via gluconeogenesis.

### Acute NRG1 treatment activates ERBB3 in liver, but not in skeletal muscle, white adipose tissue and hypothalamus of db/db mice

**Fig 5:**
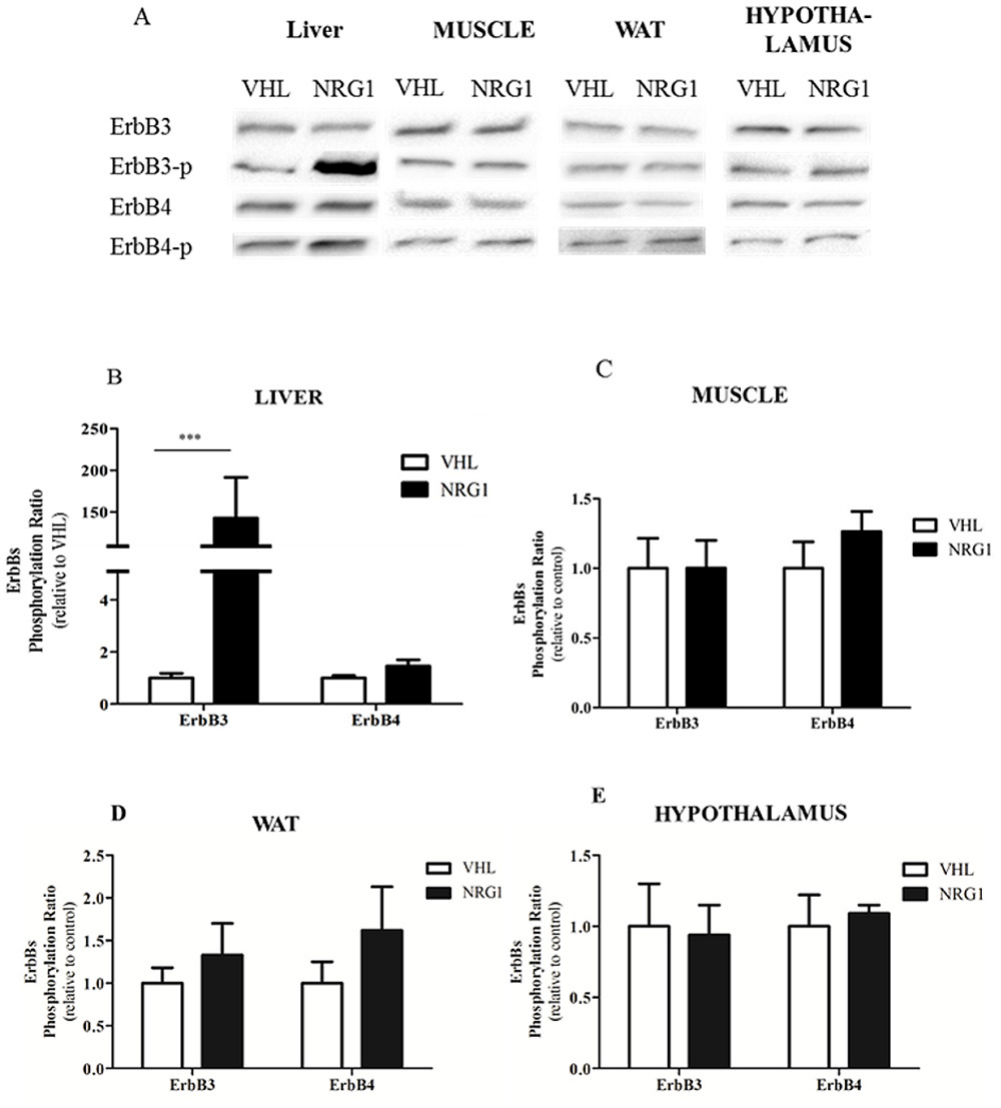
NRG1 injection activates ERBB3 in the liver but not in the gastrocnemius, WAT and hypothalamus of db/db mice. Six hour-fasted male db/db mice received an i.p. injection of vehicle (0.9% NaCl; n = 8) or NRG1 (50 μg.kg^-1^; n = 8) and were euthanized 30 minutes later. A) Western blot analysis using antibodies against total and phosphorylated (p) ERBB3 and ERBB4. ERBB3 and ERBB4 phosphorylation ratios (the ratio between phosphorylated form and total ERBB expression) in B) liver, C) skeletal muscle, D) WAT and E) hypothalamus (relative to controls, VHL). Values are the mean ± SEM. VHL: vehicle; NRG1: neuregulin 1; ***: p<0.001 compared to the VHL group.

To investigate the mechanism by which NRG1 influences glucose metabolism, liver, skeletal muscle, WAT and hypothalamus samples were collected from fasted db/db mice 30min after i.p. injection of NRG1 (50μg.kg^-1^, n = 8) or saline solution (VHL, controls, n = 8). Western blot analysis of ERBB receptor phosphorylation in the different tissue lysates indicated that acute NRG1 treatment led to increase of only ERBB3 phosphorylation and exclusively in the liver (Fig 5A). Quantification of these results showed a significantly higher phosphorylation ratio of ERBB3 (by 142 folds), but not of ERBB4, in the liver of mice treated with NRG1 compared with controls (p <0.01, Fig 5B). In contrast, the phosphorylation ratio of ERBB3 and ERBB4 were comparable in skeletal muscles (Fig 5C), WAT (Fig 5D) and hypothalamus (Fig 5E) of NRG1- and VHL-treated animals.

### Acute NRG1 treatment activates the AKT/FOXO1 pathway in liver

**Fig 6:**
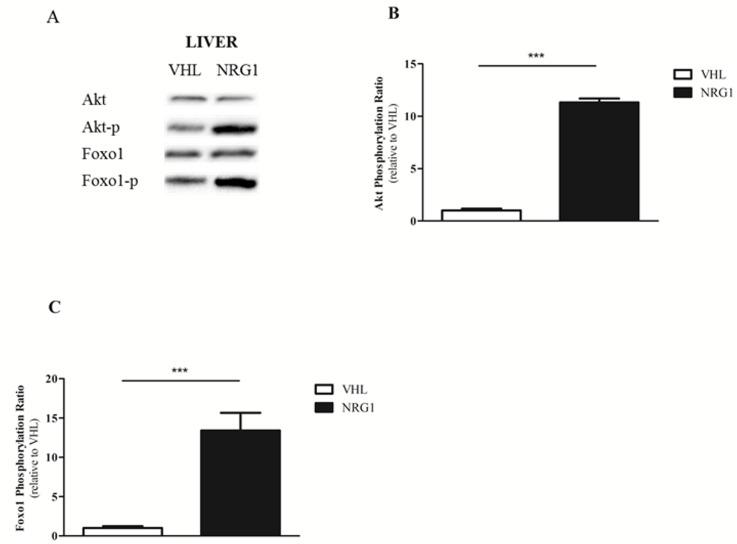
NRG1-induced activation of the AKT and FOXO1 pathways in liver of db/db mice. Six hour-fasted male db/db mice were treated with vehicle (0.9% NaCl; n = 8) or NRG1 (50 μg.kg^-1^; n = 8) by i.p. injection and were euthanized 30 minutes later. A) Western blot analysis using antibodies against AKT (total and phosphorylated on Ser473) and FOXO1 (total and phosphorylated on Ser256). Ratios of B) phosphorylated AKT (on Ser473) to total ATK and C) phosphorylated FOXO1 (on Ser256) to total FOXO1. Values shown are mean ± SEM. VHL: vehicle; NRG1: neuregulin 1; ***: p<0.001 compared to the VHL group.

FOXO1 is one of the main regulators of gluconeogenesis *in vivo*, through transcriptional activation of gluconeogenic genes [27]. AKT-induced phosphorylation of FOXO1 at Ser256 excludes FOXO1 from the nucleus, resulting in inhibition of hepatic gluconeogenesis [28, 29]. Therefore, the expression of total and phosphorylated AKT and FOXO1 was assessed by western blotting in liver samples collected from fasted db/db mice 30min after i.p. injection of NRG1 (50μg.kg^-1^, n = 8) or saline solution (VHL, controls, n = 8) (Fig 6A). Calculation of the ratio between phosphorylated protein and total protein expression following acute treatment with NRG1 or VHL showed a 11.3-fold increase of this ratio for AKT (p <0.01) (Fig 6B) and a 13.4-fold increase for FOXO1 (p <0.01) (Fig 6C) compared with control (VHL).

## Discussion

In this study, we show that acute injection of NRG1 improves glucose tolerance in db/db mice. This effect could be in part mediated through inhibition of hepatic glucose production from gluconeogenesis. We then demonstrate that chronic treatment with NRG1 also improves glucose clearance during GTT in db/db mice, suggesting that the NRG1 pathway may represent a promising therapeutic target for type 2 diabetes.

### NRG1 improves glucose tolerance in vivo

The main finding of the present study is that acute i.p. injection of NRG1 has a marked glucose-lowering effect in db/db mice. The effects of acute injection of NRG1 are moderate in basal conditions in fasted mice and do not induce a hypoglycemic response in the two hours following the injection (Fig 2). This result is particularly interesting because hypoglycemia is a common adverse effect of some type 2 diabetes medications and of insulin therapy [30, 31]. Conversely, in conditions of glucose challenge, NRG1 markedly decreases the glycemic response, reducing the glucose AUC by around 50% (Fig 3). These results suggest that NRG1 glucose-lowering effect is enhanced in conditions of hyperglycemia, which is highly attractive from a therapeutic point of view. Although some in vitro studies have already reported that NRG1 can stimulate glucose uptake in skeletal muscle tissue [15, 16, 25], our result show that NRG1 may also affect glucose homeostasis systemically, by decreasing the serum glucose response after oral glucose challenge. As glucose tolerance was also improved in db/db mice chronically treated with NRG1, our result strengthens the interest of NRG1 as a potential therapeutic agent for diabetes.

### Mode of action of NRG1 as a glucose-lowering agent

Many hypotheses can be proposed to explain the glucose-lowering effect of NRG1. The current therapies for type 2 diabetes mainly focus on three strategies: i) reducing carbohydrate absorption from the intestine, ii) increasing plasma insulin concentration, and/or iii) improving insulin sensitivity [32]. As NRG1 glucose-lowering effects are visible also in fasted state, a lowering effect on carbohydrate absorption from the intestine appears to be unlikely. Similarly, the hypothesis of an NRG1-mediated increase of insulin concentration can also be ruled out. Indeed, while acute injection of NRG1 did not modify serum insulin concentration in fasted db/db mice in basal conditions, it markedly decreased insulin level during the glucose challenge. These results were confirmed in animals chronically treated with NRG1 in which the insulin responses were decreased during the oral glucose challenge. Similarly, in rat islet-derived CRI-G1 cells, NRG1 could not stimulate insulin secretion [33]. On the other hand, the concomitant NRG1-mediated reduction of serum glucose and insulin in glucose challenge conditions suggests that NRG1 treatment improves insulin sensitivity. This hypothesis is in agreement with in vitro studies showing that incubation of L6E9 cells with NRG1 for 48h increased insulin-mediated GLUT4 translocation to the plasma membrane and glucose uptake [19]. These in vitro studies clearly designated skeletal muscle as a potential target of NRG1 in vivo, but other tissues, such as adipose tissue, liver and hypothalamus, could also play an important role.

### Skeletal muscle is not involved in NRG1-induced reduction of glucose levels in vivo

Skeletal muscle is one of the major contributors to glucose disposal and expresses the NRG1 receptors ERBB3 and ERBB4 [34, 35]. Studies in L6E9 myoblasts showed that NRG1-induced higher glucose uptake is mediated by ERBB3 and ERBB4 phosphorylation [16, 25]. In contrast to these in vitro studies, we did not detect any increase in ERBB3 and ERBB4 phosphorylation in skeletal muscle following acute injection of NRG1 in vivo. This suggests that in vivo, skeletal muscle is not involved in NRG1-induced reduction of glucose levels. Different hypothesis may explain such discrepancies. First, in the present study, we used db/db mice that are insulin-resistant and diabetics. A previous study in cardiac cells showed that palmitate treatment induces insulin-resistance and also impairs NRG1 signaling [18]. Thus, one could hypothesize that NRG1 signaling is impaired in vivo in skeletal muscle of db/db mice. Further studies are needed to assess the effects of acute injection of NRG1 on skeletal muscle in non-diabetic control mice to definitively discard this hypothesis. Second, skeletal muscle in vivo may respond differently to NRG1 treatment compared to myoblasts in vitro. This hypothesis is strengthened by the finding that ERBB expression is markedly higher in immature cultured muscle cells than in adult skeletal muscle [36]. Third, in the present study, we used the NRG1-β2 isoform, while previous works showing NRG1 effects in myoblasts used NRG1-β1 [15, 16, 25]. Different NRG1 isoforms may have distinct tissue specificity and receptor affinity. Indeed, in liver, NRG1-β is up to 100 times more active in inducing ERBB phosphorylation than NRG1-α [24].

### Liver is the main target of NRG1 in vivo

As NRG1 did not affect ERBBs in skeletal muscle, we also assessed the impact of acute NRG1 treatment on ERBB phosphorylation in hypothalamus, adipose tissue and liver, three tissues potentially involved in glucose metabolism regulation. Acute injection of NRG1 did not change ERBB3 and ERBB4 phosphorylation ratio in adipose tissue and hypothalamus, indicating that these tissues are not the main NRG1 targets in vivo. Conversely, we observed a huge increase in hepatic ERBB3 phosphorylation following NRG1 injection. This indicates that liver is the main NRG1 target. Liver is very involved in the control of glucose homeostasis in vivo [37]. Insulin can enhance glucose hepatic storage and utilization and inhibit glucose synthesis and release [38]. The finding that NRG1 injection markedly reduced serum glucose level and increased blood lactate concentration induced by pyruvate challenge strongly suggests that NRG1 inhibits gluconeogenesis, which mainly occurs in the liver. During pyruvate challenge, this would result in pyruvate conversion to lactate via lactate dehydrogenase (LDH), therefore explaining the increased lactate concentration. We cannot rule out the possibility that NRG1 might also affect pyruvate oxidation. However, this last effect could only explain the increased lactate level during PTT, but not the decrease of serum glucose level. Biochemical experiments also strengthen the hypothesis that NRG1 might inhibit liver gluconeogenesis. FOXO1 is a key regulator of hepatic glucose production *in vivo* [39] and a major regulator of gluconeogenesis [40, 41]. Insulin induces AKT-dependent phosphorylation of FOXO1, resulting in FOXO1 nuclear exclusion and down-regulation of glucose-6-phosphatase (G6Pase) and phosphoenolpyruvate carboxy-kinase (PEPCK), two rate-limiting enzymes of hepatic gluconeogenesis [42–46]. Here, we show that acute NRG1 injection strongly increases AKT phosphorylation on Ser473 and FOXO1 phosphorylation on Ser256. These results suggest that NRG1 induces insulin-like effects on hepatic glucose production *in vivo*. However, we detected NRG1 effects on glucose homeostasis during GTT and PTT as early as 30 minutes after NRG1 injection. This suggests that NRG1-mediated FOXO1 phosphorylation cannot fully account for the rapid reduction of serum glucose level following NRG1 injection. Finally, it was previously reported that insulin-mediated AKT phosphorylation on Ser473 is impaired in liver of db/db mice [47]. Conversely, we show that NRG1 can strongly phosphorylate AKT at Ser473 and FOXO1 at Ser256 in db/db mice, suggesting that the NRG1 pathway in liver is functional also in conditions of insulin resistance. This observation could open interesting therapeutic avenues.

### Therapeutic potential of NRG1

Type 2 diabetes is one of the most important public health challenges worldwide. Although therapeutic options have greatly increased over the past 20 years, current medications are considered far from ideal and cause various side effects [48]. Specifically, insulin therapy, which is commonly used in type 2 diabetes, is frequently associated with hypoglycemia events and weight gain [49]. Therefore, research programs to evaluate new therapeutic strategies for type 2 diabetes are fundamental. We show that acute injection of NRG1 improves glucose tolerance, by a mechanism involving inhibition of hepatic glucose production. Mice chronically treated with NRG1 also show improved glucose tolerance and we previously demonstrated that this treatment does not affect body weight in db/db mice [50]. Our study proposes some explanation on the mechanism of NRG1-induced lower serum glucose level; however, further studies are needed to explore the mechanisms involved in chronic treatment with NRG1. It could be also interesting to assess the effect of NRG1 chronic treatment on glycosylated hemoglobin (HBA1c) using different animal pre-clinical models, such as diet-induced insulin-resistance. If the potential of NRG1 to improve glycemic control in diabetic state is confirmed, strategies to enhance the quite short half-life of circulating NRG1 (about 30min) [51] could be considered.

In conclusion, our results show for the first time that an acute injection of NRG1 decreases serum glucose responses after oral glucose challenge in db/db mice. This effect may be due to hepatic gluconeogenesis inhibition. Chronic NRG1 treatment may also improve glucose tolerance in db/db mice, suggesting that the NRG1 pathway may represent a promising therapeutic target in conditions of insulin resistance.
